# A Novel Capillary Electrophoresis-Based High-Throughput Multiplex Polymerase Chain Reaction System for the Simultaneous Detection of Nine Pathogens in Swine

**DOI:** 10.1155/2017/7243909

**Published:** 2017-06-11

**Authors:** Xu-long Wu, Lu Xiao, Hua Lin, Miao Yang, Shi-jie Chen, Wei An, Yin Wang, Xue-ping Yao, Ze-xiao Yang, Zi-zhong Tang

**Affiliations:** ^1^College of Veterinary Medicine, Sichuan Agricultural University, Chengdu 611130, China; ^2^Inspection and Quarantine Technical Center, Sichuan Entry-Exit Inspection and Quarantine Bureau, Chengdu 610041, China; ^3^Key Laboratory of Animal Disease and Human Health of Sichuan Province, Chengdu 611130, China; ^4^College of Life Science, Sichuan Agricultural University, Ya'an 625014, China

## Abstract

Here we aimed to develop a capillary electrophoresis-based high-throughput multiplex polymerase chain reaction (PCR) system for the simultaneous detection of nine pathogens in swine. Nine pairs of specific primers and a set of universal primers were designed; the multiplex PCR was established. The specificity and cross-reactivity of this assay were examined, and the detection limit was determined using serial 10-fold dilutions of plasmids containing the target sequences. The assay was further tested using 144 clinical samples. We found that the nine specific amplification peaks were observed, and the assay had a high degree of specificity, without nonspecific amplification. The simultaneous detection limit for the nine viruses reached 10000 copies *μ*L^−1^ when all of the premixed viral targets were present. Seventy-seven of the clinical samples tested positive for at least one of the viruses; the principal viral infections in the clinical samples were porcine circovirus type 2 and porcine reproductive and respiratory syndrome virus. This approach has much potential for further development of high-throughput detection tools for the diagnosis of diseases in animals.

## 1. Introduction

With advances in pig-breeding technologies and swine production, single or mixed (multiple) infections are becoming increasingly common on pig farms [1, 2]. The major viral pig pathogens are pseudorabies virus (PRV), Japanese encephalitis virus (JEV), classic swine fever virus (CFSV), porcine circovirus type 2 (PCV-2), porcine reproductive and respiratory syndrome virus (PRRSV), porcine parvovirus (PPV), foot-and-mouth disease virus (FMDV), porcine epidemic diarrhea virus (PEDV), and transmissible gastroenteritis virus (TGEV). These viruses cause diseases in pigs with high morbidity and mortality and are a major problem for the swine industry [3]. The similar symptoms caused by each of these virus infections make diagnosis difficult [4]. In addition, PRRSV, PRV, CSFV, and PCV-2 can also cause immunosuppression [5], creating conditions suitable for secondary infection with other pathogens or further complications. Therefore, there is a need for an effective, rapid, and high-throughput method for the simultaneous diagnosis of these viral pathogens.

The QIAxcel capillary electrophoresis system (QCES) is an accurate, automated DNA sizing system, which is widely applied in detection due to its automation, superior accuracy, and ease of use [6]. The QCES utilizes cartridges comprising an array of 12 capillaries prefilled with gel polymers, thus minimizing manual handling [7]. These short capillaries can detect DNA fragments between 15 base pairs (bp) to 10 kilobases (kb) and provide a resolution as high as 3–5 bp. Thus, amplification products can be analyzed in a 96-well plate in one experimental running, and usually, 12 products are analyzed in approximately 10–15 min. Furthermore, the amplicon sizes can be analyzed automatically and presented as peaks at the end of the detection procedure [8]. In recent years, the QCES has been increasingly applied to high-throughput nucleic acid analysis applications, such as pathogen detection [9], genotyping [10], discrimination of the alleles [11, 12], and species identification [8, 13].

Here, we describe a novel multiplex PCR-QCES assay for the simultaneous detection of nine pathogens in swine. Based on our findings, we recommend that routine testing laboratories adopt this approach, which will allow users to process more samples in less time compared to existing assays and platforms.

## 2. Materials and Methods

### 2.1. Positive Strains and Clinical Sample

Positive viruses of PRV (Bartha-K61, HB-98 strain), JEV (SA14-14-2 strain), CSFV (HCLV strain), PCV-2 (LG, ZJ/C strain), PRRSV (CH-1R, R98, HUN4, JXA1-R, CH-1a strain), PPV (WH-1, CP-99 strain), FMDV (O, A, AsiaI), TGEV, and PEDV (CV777 strain) were obtained from commodity vaccines or provided by the Animal Quarantine Laboratory, Sichuan Agricultural University. As negative controls, rotavirus (RV), bovine viral diarrhea virus (BVDV),* Salmonella*,* Pasteurella multocida* (Pm), methicillin-resistant* Staphylococcus aureus* (MRSA),* Haemophilus parasuis* (HPS), and* Streptococcus suis* were provided by the Animal Quarantine Laboratory, Sichuan Agricultural University.

The 144 clinical samples (including 62 of visceral tissues, 14 of abortus, 23 of semen, and 45 of blood) were collected from pig farms in Sichuan, China, between 2016 and 2017, provided by the Animal Quarantine Laboratory, Sichuan Agricultural University, and the Inspection and Quarantine Technical Center, Sichuan Entry-Exit Inspection and Quarantine Bureau. Animal welfare and experimental procedures were carried out in accordance with the Guide for the Care and Use of Laboratory Animals and were approved by the animal ethics committee of Sichuan Agricultural University.

### 2.2. Primer Design

Nine pairs of specific primers were designed in a highly conserved region and were evaluated using the National Center for Biotechnology Information (NCBI) Primer-Blast and Primer Premier software. Each specific primer was fused to a labeling sequence at its 5′-end (defined as a chimeric specific primer). One additional pair of universal primers was designed to recognize the label sequences. The primer sequences, their target genes, and the size of the amplicons are summarized in Table 1. All primers were synthesized by Invitrogen™ (Shanghai, China).

### 2.3. Preparation of Nucleic Acid and Plasmids

The viral genomic DNA or RNA was extracted using the QlAamp Viral DNA/RNA Mini Kit (QIAGEN, Hilden, Germany). RNAs were reverse-transcribed using the PrimeScript™ RT Reagent Kit (TaKaRa BIO Inc., Dalian, China).* Salmonella*, Pm, MRSA, HPS, and* S. suis* were cultured, and the genomic DNA was extracted using TaKaRa MiniBEST Bacteria Genomic DNA Extraction Kit (TaKaRa). All DNA and cDNA were stored at −20°C.

The target genes of the nine porcine viral pathogens were amplified using their specific primer pair. The amplified products were purified using the TaKaRa MiniBEST Agarose Gel DNA Extraction Kit (TaKaRa BIO Inc., Dalian, China). The purified DNA was ligated into the pMD19-T vector, and the ligated constructs were transformed into* E. coli* DH5a cells cultured in the presence of ampicillin (100 *μ*g/mL). The recombinant plasmid construct was confirmed by DNA sequencing (Life Technologies Inc., Shanghai, China), and the sequence data were analyzed using DNASTAR software and compared with the corresponding sequence data in GenBank.

### 2.4. Development of the Mono-PCR Assay

Mono-PCR assays were developed with DNA/cDNA to evaluate the specificity of each pair of primers and to determine the amplicon size of each target region. The mono-PCR assay contained 12.5 *μ*L of 2x Ex Taq, 1 *μ*L of DNA/cDNA, 20 *μ*M each of the universal primer, and 1.25 *μ*M each of the chimeric specific primer, in a final reaction volume of 25 *μ*l. The PCR was performed under the following conditions: 94°C for 3 min, followed by 15 cycles of 94°C for 30 s, 60°C for 60 s, and 72°C for 1 min and 30 cycles of 94°C for 15 s, 5°C for 30 s, 72°C for 30 s, and 72°C for 10 min. The amplification products were analyzed by QCES and confirmed by DNA sequencing after the amplification cycles.

### 2.5. Establishing and Optimizing the Multiplex PCR-QCES Assay

The assay conditions were optimized by varying one parameter at a time. The final concentrations of the nine specific chimeric primers were optimized from 20 to 100 nmol/L in 25 *μ*L reactions, selecting the optimal proportion of primers. The final concentrations of the universal primers were optimized from 200 to 800 nmol/L and the annealing temperature was optimized from 52 to 62°C. The multiplex PCR assay was performed using the QIAGEN Multiplex PCR Kit (QIAGEN) and the products were analyzed by QCES.

### 2.6. Separation by Capillary Electrophoresis and Fragment Analysis

The PCR products were placed directly into the QCES test platform and analyzed using the QIAxcel DNA High-Resolution Kit (QIAGEN). A custom alignment marker of 15–600 bp was run simultaneously with the samples, and the QX DNA size marker of 25–500 bp was used for size estimation. The reaction products were analyzed using the OM500 method at 5 kV and a 500 s separation time. The alignment marker was injected at 4 kV for 20 s and samples at 5 kV for 10 s. The QCES automates the process of detecting and measuring the size of the PCR-amplified DNA fragments.

### 2.7. Evaluating the Detection Limit of the Multiple PCR by QIAxcel Assay

Nine target genes were amplified: the gB genes of PRV, N genes of PRRSV, E genes of JEV, E2 gene of CSFV, ORF1 genes of PCV-2, VP1 genes of PPV, 3D genes of FMDV, N genes of PEDV, and N genes of TGEV. The recombinant plasmids of these nine target genes were constructed and mixed in equal proportions after quantitation using NanoDrop 2000 (Thermo Fisher Scientific Inc., DE, USA). The copy number of the plasmid was calculated according to the formula [copies/*μ*L = 6 × 10^23^ × DNA concentration (g/*μ*L)/molecular weight (g/mol)] [14]. Serial 10-fold dilutions of the plasmid mixture from 10^7^ copies/*μ*L to 10^2^ copies/*μ*L were performed to evaluate the detection limit of this assay for simultaneous detection of the nine viruses. The detection limit of mono-PCR was also confirmed using the serial dilutions of the plasmid, respectively. The detection limit was determined as the last serial dilution that gave a positive result.

### 2.8. Cross-Reactivity Assay

To test the cross-reactivity of the multiplex PCR-QCES assay, different combinations of DNA/cDNA including the nine target pathogens and other negative control pathogens (RV, BVDV,* Salmonella*, Pm, MRSA, HPS, and* S. suis*) were tested. The products were analyzed by QCES (as described above), and the specificity of the amplicons was confirmed by DNA sequencing (Life Technologies Inc.).

### 2.9. Application of Clinical Samples

A total of 144 clinical samples including visceral tissues, abortus, semen, and blood were collected from pig farms in Sichuan area, China, between 2016 and 2017. All of the samples were tested using the multiple PCR-QCES, and the positive clinical samples were reconfirmed by traditional PCR/RT-PCR using the same nine sets of specific primers and DNA sequencing.

## 3. Results

### 3.1. Development of the PCR-QCES Assay

**Figure 1:**
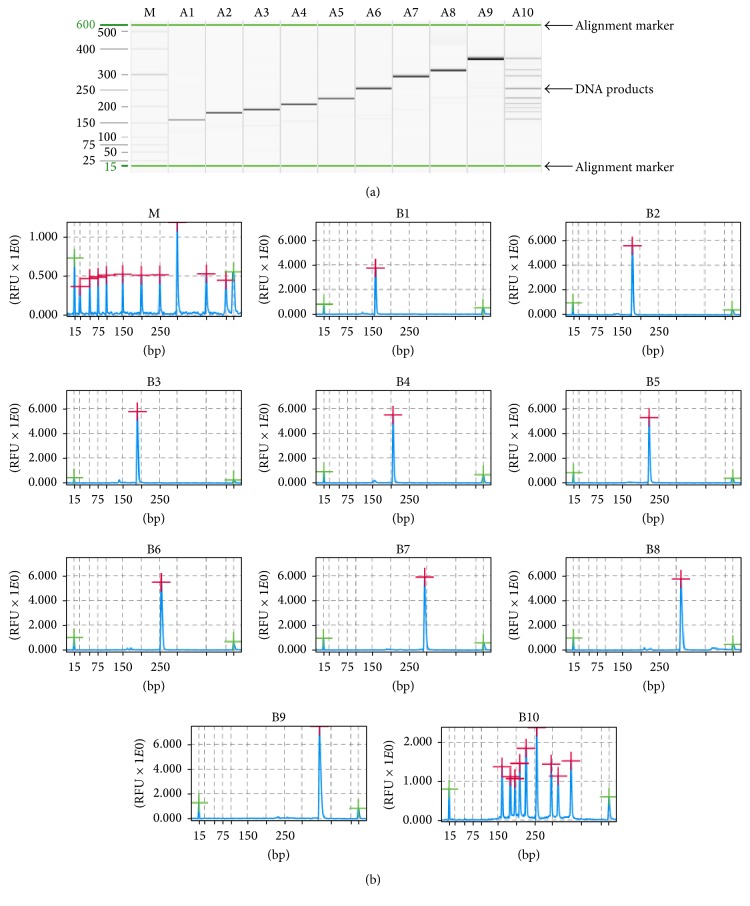
The PCR amplification products were analyzed using the QIAxcel system. The results of QIAxcel gel image (a) and amplification peaks (b). M: the QIAxcel size marker, 25–500 bp; Lanes A1–A10 and lanes B1–B10: the assay was performed with PRV, PRRSV, JEV, FMDV, CSFV, PCV-2, TGEV, PEDV, PPV, and nine mixed DNA, respectively.

Each pair of chimeric specific primers was initially tested in a mono-PCR-QCES assay to determine its amplicon size. The multiplex PCR-QCES assay was established after optimizing the reaction conditions. The multiplex PCR assay was performed using the QIAGEN Multiplex PCR Kit (QIAGEN) in a 25 *μ*L volume containing 12.5 *μ*L of 2x QIAGEN Multiplex PCR Master Mix, 5 *μ*L of 5x Q-Solution, 2 *μ*l of template, and primers (final concentrations of 50 nM for PRV, JEV, PEDV, PRRSV, CSFV, PCV-2, and PPV; 100 nM for FMDV and TGEV; and 400 nM of universal primer). The PCR was performed under the following conditions: 95°C for 15 min, followed by 15 cycles of 94°C for 30 s, 60°C for 90 s, and 72°C for 90 s and 35 cycles of 94°C for 30 s, 51°C for 30 s, 72°C for 60 s, and 72°C for 10 min. The QCES analysis for the nine specific porcine pathogens is shown in Figure 1. The results are shown as a gel-like image, as well as an electropherogram (Figure 1(a)). All of the viruses could be detected without nonspecific amplification, and the expected amplification stripes were observed, with peaks corresponding to the expected amplicon size (Figure 1(b)). The amplification products were confirmed by DNA sequencing and comparison to sequences deposited in GenBank. The amplified sequences had greater than 99% homology with the targeted viruses.

### 3.2. Cross-Reactivity Assay

**Figure 2:**
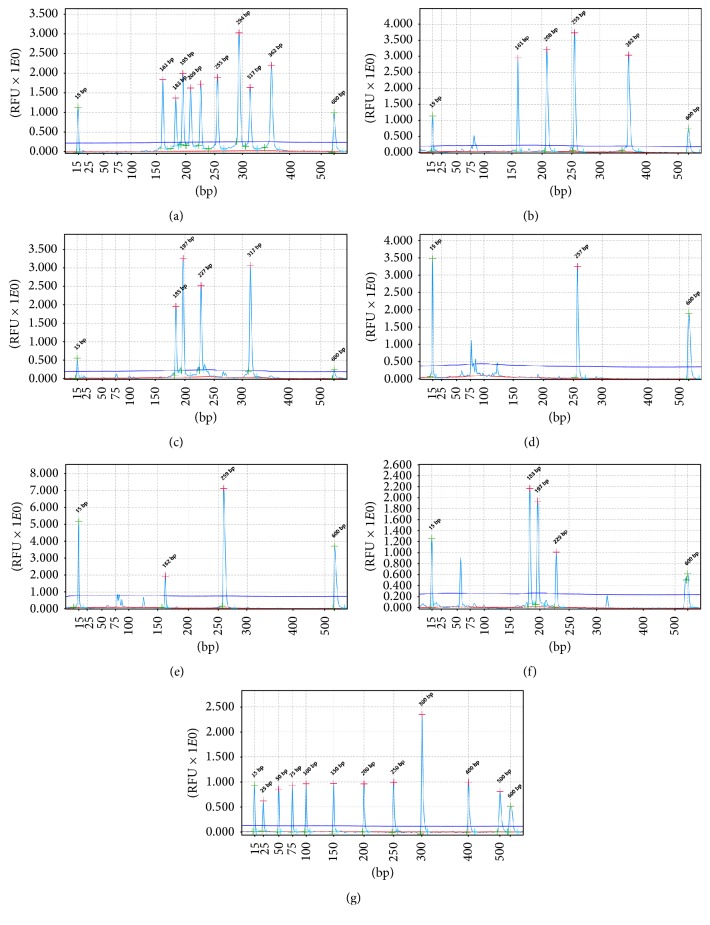
Cross-reactivity assay and detection of clinical samples. Different combinations of pathogens DNA were used as templates (a–c); all of the specific amplification peaks were observed, presenting the gene-specific target amplicon without cross-amplification. The detection results of clinical samples (d–f). (a) The templates were nine target pathogens. (b) The templates were PRV, FDMV, PCV-2, PPV, RV, BVDV,* Salmonella*, Pm, MRSA, HPS, and* S. suis*. (c) The templates were PRRSV, JEV, CSFV, PEDV, RV, BVDV,* Salmonella*, Pm, MRSA, HPS, and* S. suis*. (d) The sample was PCV-2 positive. (e) The sample was PRV and PCV-2 positive. (f) The sample was PRRSV, JEV, and CSFV positive. (g) QX DNA size marker, 25–500 bp.

To evaluate cross-reactivity, we subjected different combinations of DNA/cDNA to our multiple PCR-QCES assay. All of the viruses could be detected without nonspecific amplification or cross-reactivity (Figures 2(a)–2(c)). The amplicons were sequenced, confirming that all of the reactions produced specific amplifications. Similar results were obtained when these reactions were repeated three times, indicating that this method has a high degree of specificity for simultaneous and rapid detection of nine porcine pathogens.

### 3.3. Evaluation of Multiple PCR-QCES Detection Limits

**Figure 3:**
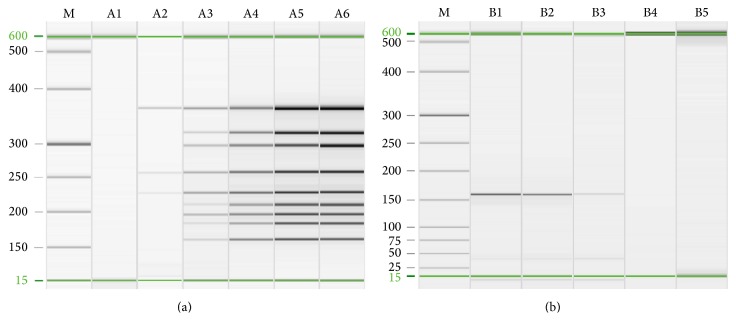
Sensitivity test of multiple PCR and PRV mono-PCR by QIAxcel; the detection limit of multiple PCR-QCES achieved approximately 10^4^ copies/*μ*L when all nine templates were present (a) and the detection limit of PRV mono-PCR achieved approximately 10^2^ copies/*μ*L (b). M: QX DNA size marker, 25–500 bp. Lanes A1–A6: the template mixtures were 10^2^ copies/*μ*L to 10^7^ copies/*μ*L, respectively; lanes B1–B5: 10^4^ copies/*μ*L to 10^0^ copies/*μ*L.

The recombinant plasmids of nine target genes were quantified and mixed in equal proportions. The plasmid mixtures were serially diluted 10-fold and used to measure the detection limit of the method. By this approach, we found that the detection limits for CSFV, PCV-2, and PPV were 10^3^ copies/*μ*L, which were higher than those for other target viruses. When all nine templates were present, the detection limit of the multiple PCR-QCES method was approximately 10^4^ copies/*μ*L (Figure 3(a)). Primer dimers did appear as the template concentration reduced, but these were usually below 100 bp (data not shown), and it did not affect the result. In addition, the detection limit of mono-PCR for PRV, CSFV, JEV, PEDV, TGEV, PCV-2, and PPV reached 10^2^ copies/*μ*L, which was higher than that for PRRSV and FMDV of 10^3^ copies/*μ*L (Figure 3(b); only the data of PRV was shown).

### 3.4. Application of Clinical Samples

A total of 144 clinical samples, including 62 of visceral tissues, 14 of abortus, 23 of semen and 45 of blood, were tested using the multiplex PCR-QCES. Seventy-seven clinical samples tested positive (53.47%) for at least one virus (Table 2). Some results of clinical samples detection were shown in Figures 2(d)–2(f). The positive clinical samples were reconfirmed by traditional PCR/RT-PCR, and the detection results were consistent. The visceral tissues and abortus produced noticeably higher detection rates than the semen and blood samples, which was likely because the tissue samples were collected from dead pigs and aborted fetuses (the semen and blood were collected from pigs used for breeding). PRRSV and PCV-2 infections were detected more often than the other viruses, and coinfection with two or more viruses was detected in 40 (51.95%) specimens. These data suggest that multiple pathogenic mixed infections (especially PCV-2 and PRRSV infections) are a serious problem for swine farmers.

## 4. Discussion

Multiple pathogenic mixed infections have become increasingly commonplace and cause serious economic losses to the swine industry. Here, we found that mixed infections are a serious problem, with mixed infections detected in 40 (51.95%) of 77 positive samples. PRRSV and PCV-2 infections were detected more often than the other tested viruses. These infections might cause immunosuppression within the swinery and create conditions for secondary infection with other pathogens or further complications. Based on our findings, we propose that further actions are needed to prevent mixed infections, especially of PCV-2 and PRRSV. In addition, because of the evolution and variation of viruses, as well as the typical conditions of intensive pig production, it is common for pigs to be simultaneously infected with two or more viral pathogens, which can induce more severe clinical syndromes and lesions [15, 16]. The conventional laboratory diagnosis methods used for the separate detection of each virus (e.g., conventional PCR and real-time PCR) can be time-consuming and expensive. Multiplex PCR has the potential to produce considerable time and cost savings, as well as reducing the number of samples required, which is particularly important when sample materials are limited [1].

The QIAxcel, an automated 12-channel capillary electrophoresis system, is an alternative to agarose gel electrophoresis [17]. Agarose gel electrophoresis is the most widely used DNA detection/sizing system and is advantageous because of its simplicity and low cost. However, agarose gel electrophoresis is labor-intensive and time-consuming and exposes users to a hazardous carcinogen (ethidium bromide) [6]. The QCES does not require the preparation of gels nor nucleic acid dye and is, therefore, being increasingly applied. The QCES is advantageous because it is suited to automation and is safe. The QCES can be equipped with commercial kits that differ in resolution. The highest available resolution is between 3 and 5 bp, allowing accurate fragment analysis. Using preprogrammed markers, this system can determine the size of the amplified products. In addition, the QCES uses minute amounts of DNA through electrokinetic injection. Also, the samples are retained for downstream procedures, such as sequencing. The QCES is also able to quantify the relative intensities of the amplification products [18].

In our hands, the estimated size of the amplifications products would sometimes deviate by about 5 bp between batches. However, this remains more accurate than agarose gel electrophoresis approaches. In addition, the PCR products need to be transferred to the QCES test platform to be analyzed after PCR amplification; in this process the high sensitivity detection method may lead to cross-contamination. Therefore, to achieve high-throughput gene amplification and product testing, integration and automation may be the future direction of development.

Primer design was key to the success of the method described here. The PCR amplifications were done using a novel target-enriched multiplex PCR (Tem-PCR) approach [19, 20]. This reduced the occurrence of nonspecific amplification in the reaction, ultimately decreasing the probability of false negative results. Although mixing multiple primers did lead to the formation of primer dimers when the template DNA concentrations were low, the small dimers produced here did not affect the assay outputs.

Based on our findings, we propose that this assay should be considered as a novel and effective method applicable to smaller reference and regional laboratories and that its performance is superior to manual gel electrophoresis PCR fragment separation, with better accuracy and shorter detection times. We believe that combining and assembling detection kits for practical applications, such as what has been described here, is now necessary and pertinent and has the potential to enhance rapid responses for prompt treatment and control in the swine industry.

## 5. Conclusion

Here we describe a novel, rapid multiplex PCR using the QIAxcel system that is able to detect PRV, CSFV, FMDV, JEV, PCV-2, PRRSV, PEDV, TGEV, and PPV infections in swine. This assay is sensitive, specific, and high-throughput. Compared to current methods, this approach is more convenient, efficient, and reliable for laboratory diagnosis of mixed infections in porcine clinical specimens and improves detection efficiency. We propose that this method can be used in epidemiological investigations and laboratory identification of clinical isolates.

## Figures and Tables

**Table 1:** Primers information of QIAxcel assay.

Name	Sequence^a^ (5′-3′)	Gene	Size range (bp)
PRV	F: AGGTGACACTATAGAATAAGGGGTTGGACAGGAAGGACA	gB	161
	R: GTACGACTCACTATAGGGACATCGCCAACTTCTTCCAGG		
PRRSV	F: AGGTGACACTATAGAATACATCGCCCAACAAAACCAGTCC	N	183
	R: GTACGACTCACTATAGGGAACGACAGACACAATTGCCGCTC		
JEV	F: AGGTGACACTATAGAATATGGCTCTATTGGAGGGGTCT	E	197
	R: GTACGACTCACTATAGGGAAATTGATCGGTCTCGTGCGT		
FMDV	F: AGGTGACACTATAGAATACCGACAAAAGCGACAAAGGTT	3D	211
	R: GTACGACTCACTATAGGGAATCAACTTCTCCTGTATGGTCCC		
CSFV	F: AGGTGACACTATAGAATATGGCAAATGAGACAGGTTACAGA	E2	229
	R: GTACGACTCACTATAGGGATCCTTACAGGTCCCGCACTA		
PCV-2	F: AGGTGACACTATAGAATAGGAAGAAGCGGACCCCAACC	ORF1	255
	R: GTACGACTCACTATAGGGAAGCGGGCACCCAAATACCA		
TGEV	F: AGGTGACACTATAGAATAGGAACTTATGTCCGAGAGACTTTG	N	295
	R: GTACGACTCACTATAGGGAGGATTCATTATTAGCACCACGACT		
PEDV	F: AGGTGACACTATAGAATAACTAATAAGGGGAATAAGGACCA	N	317
	R: GTACGACTCACTATAGGGAAACAATCTCAACTACACTGGGGA		
PPV	F: AGGTGACACTATAGAATAATACTTGGGGGAGGGCTTGG	VP1	363
	R: GTACGACTCACTATAGGGATTGGTGGTGAGGTTGCTGAT		
SP	F: AGGTGACACTATAGAATA		
	R: GTACGACTCACTATAGGGA		

^a^Sequence: the underline means label sequences.

**Table 2:** Results of clinical samples detection.

Sample type/pathogens^a^	Number of positive cases	Positive^b^ rate% (positive/sample)
*The sample type*		
Visceral tissues	53	85.48% (53/62)
Abortus	11	78.57% (11/14)
Semen	4	17.39% (4/23)
Blood	9	20.00% (9/45)
Total	77	53.47% (77/144)
*Pathogens*		
PRV	2	2.60%
PRRSV	8	10.39%
JEV	3	3.90%
FMDV	1	1.30%
CSFV	4	5.19%
PCV-2	12	15.58%
PEDV	5	6.49%
PPV	2	2.60%
PRV + PCV-2	5	6.49%
PRV + CSFV	2	2.60%
PCV-2 + PRRSV	17	22.08%
PCV-2 + CSFV	6	7.79%
PEDV + TGEV	3	3.90%
PRRSV + CSFV	2	2.60%
PCV-2 + PRRSV + CSFV	3	3.90%
CSFV + JEV + PRRSV	1	1.30%
FMDV + PRV + PCV-2 + PPV	1	1.30%

^a^PRV: pseudorabies virus; CSFV: classic swine fever virus; JEV: Japanese encephalitis virus; PCV-2: porcine circovirus type 2; PRRSV: porcine reproductive and respiratory syndrome virus; PPV: porcine parvovirus. FMDV: foot-and-mouth disease virus. PEDV: porcine epizootic diarrhea virus. TGEV: transmissible gastroenteritis virus. ^b^At least one kind of virus was tested positive.
